# Cardiogenic and chronobiological mechanisms in seizure-induced sinus arrhythmias

**DOI:** 10.1371/journal.pcbi.1013318

**Published:** 2025-07-16

**Authors:** Pan Li, Sangbo Lee, Kwang-Yeon Choi, Jonathan E. Rubin, Jae Kyoung Kim

**Affiliations:** 1 Biomedical Mathematics Group, Pioneer Research Center for Mathematical and Computational Sciences, Institute for Basic Science, Daejeon, Republic of Korea; 2 Division of Pediatric Neurology, Department of Pediatrics, Severance Children’s Hospital, Epilepsy Research Institute, Yonsei University College of Medicine, Seoul, Republic of Korea; 3 Department of Psychiatry, College of Medicine, Chungnam National University, Daejeon, Republic of Korea; 4 Department of Mathematics, University of Pittsburgh, Pittsburgh, Pennsylvania, United States of America; 5 Department of Mathematical Sciences, KAIST, Daejeon, Republic of Korea; 6 Department of Medicine, College of Medicine, Korea University, Seoul, Republic of Korea; University of Cincinnati College of Medicine, UNITED STATES OF AMERICA

## Abstract

Seizure-induced cardiac arrhythmias, such as ictal (during seizure) or postictal (post-seizure) sinus arrhythmias, are potential triggers for sudden unexpected death in epilepsy. Traditionally, these arrhythmias have been attributed to changes in autonomic balance during ictal or postictal phases, as per the neurogenic mechanism. However, it remains unclear if these arrhythmias may involve intrinsic cardiogenic mechanisms. Furthermore, while circadian and sleep-wake patterns influence both neurogenic and cardiogenic mechanisms, a direct mechanistic link to seizure-induced arrhythmias remains to be established. In this study, we utilized a mathematical model of mouse sinoatrial nodal cell pacemaking and an autonomic clamping protocol, to dissect neurocardiogenic mechanisms in seizure-induced sinus arrhythmias and to test the hypothesis that circadian and sleep-wake rhythms directly modulate cellular susceptibility to these arrhythmias. Our simulations revealed that, in the context of altered autonomic levels associated with seizure progression, diverse seizure-induced sinoatrial nodal cell firing patterns during ictal or postictal phases can be triggered directly by intrinsic cardiac dynamics, without the need for dynamical changes in within-phase autonomic activities. This finding highlights the distinct roles of neurogenic and cardiogenic mechanisms in shaping sinoatrial nodal cell firing patterns, challenging the predominance of the neurogenic mechanism. This neurocardiogenic framework also successfully captures distinct circadian and vigilance state patterns of seizure-induced arrhythmias. Specifically, while daytime sleep predisposed sinoatrial nodal cells to postictal sinus arrhythmias, nighttime wakefulness promotes ictal sinus arrhythmias. However, these circadian patterns can be disrupted when sleep-wake cycles are decoupled from circadian rhythms, supporting the hypothesis that sleep-wake patterns can directly be a key determinant of seizure-induced sinus arrhythmias. Our findings may facilitate the development of novel therapeutic strategies for managing the risk of sudden unexpected death in epilepsy.

## Author summary

Seizure-induced sinus arrhythmias are potential triggers for sudden unexpected death in epilepsy. Traditionally, these arrhythmias have been considered as secondary effects resulting from changes in autonomic balance. However, our research suggests that the heart itself may play a more active role. Using a mathematical model of pacemaker cells in the mouse heart, we found that seizure-induced sinus arrhythmias can occur without transient autonomic changes during or after seizures. We also investigated the influence of sleep-wake patterns and circadian rhythms on these arrhythmias. Our findings indicate that daytime sleep increases the risk of post-seizure arrhythmias, while nighttime wakefulness makes arrhythmias more likely during a seizure. Importantly, these circadian patterns can be disrupted when sleep-wake cycles are misaligned with circadian rhythms, underscoring the critical role of sleep-wake patterns in shaping seizure-induced sinus arrhythmias. These mechanistic insights may help pave the way for developing novel strategies to manage the risk of sudden unexpected death in epilepsy.

## Introduction

Sudden Unexpected Death in Epilepsy (SUDEP; Table 1) is the leading cause of death in patients with epilepsy [1,2] and arises from complex, interrelated disturbances in the autonomic, cardiac and respiratory systems, typically initiated by seizures [3]. Seizures can disrupt autonomic output, leading to an autonomic imbalance that predisposes the heart to arrhythmias [4–6]. At the same time, seizure activity may impair normal respiratory function, resulting in central apnea and subsequent hypoxemia, which further destabilizes autonomic control [7]. This disruption in respiratory regulation may also exacerbate cardiac dysfunction by facilitating the onset of arrhythmias. In this context, seizure-induced cardiac arrhythmias, driven by both neurogenic and respiratory factors, may represent a tipping point in the cascade of events compromising autonomic, respiratory, and cardiac function during an epileptic seizure event [8]. They can reciprocally further impair the neuro-respiratory system by reducing cardiac output, disrupting autonomic feedback loops, and promoting hypoxia, thereby perpetuating a self-reinforcing cycle that ultimately contributes to SUDEP [9].

**Table 1:** Definitions of non-standard abbreviations.

Abbreviations	Definitions
ANS	Autonomic nervous system
AP	Action potential
BPM	Beats per minute
BT	Body temperature
CRBT	Circadian rhythm of body temperature
FR	Firing rates
HCN	Hyperpolarization-activated cyclic nucleotide-gated channel
I_CaL_	L-type Ca current
I_CaT_	T-type Ca current
I_i_, I_p_	Irregular firing during ictal and postictal phases, respectively
I_KACh_	Muscarinic K current
LCR	Local circadian rhythmicity
N_i_, N_p_	No-firing during ictal and postictal phases, respectively
P_i_, P_p_	PNA scaling factors during ictal and postictal phases, respectively
PNA	Parasympathetic nervous activity
R_i_,R_p_	Rhythmic firing during ictal and postictal phases, respectively
S, P	Preictal SNA and PNA levels, respectively
SANC	Sinoatrial nodal cells
S_i_, S_p_	SNA scaling factors during ictal and postictal phases, respectively
SNA	Sympathetic nervous activity
SUDEP	Sudden unexpected death in epilepsy
ZT	Zeitgeber time
τ, τ_p_	Time durations of ictal and postictal ramping phases, respectively

**Fig 1:**
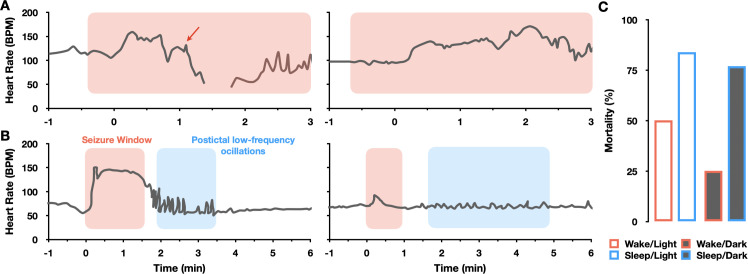
Clinical and experimental measurements of seizure-induced sinus arrhythmias and mortality. **(A-B)** Representative clinical heart rate recordings from patients with epilepsy, capturing patterns before, during (pink blocks), and after seizures. **(A)** Ictal heart rate patterns in infants experiencing apneic seizures, showing mixed sinus tachycardia and bradycardia (left), or tachycardia alone (right) [16]. The red arrow marks the onset of progressive sinus bradycardia during the seizure. Data from Maruyama et al, Pediatric Neurology, 127: p52, 2022. **(B)** Postictal low-frequency heart rate oscillations were observed in adult patients with partial epilepsy [22]. Before the seizure, heart rate maintains a respiratory sinus rhythm at 0.3Hz. During the seizure, heart rate elevates, and it subsequently transitions into transient low-frequency heart rate oscillations at 0.13Hz (left) and 0.07Hz (right) (blue blocks). Data from Al-Aweel et al, Neurology, 53: p1591-1592, 1999. **(C)** Circadian and sleep-wake patterns of mortality rates in adult mice following maximal electroshock-induced seizures [28]. Fatalities are predominantly observed in seizures that occur during sleep, both under dark and light conditions. Data from Purnell et al, Journal of Neurophysiology, 118 [5]: p2594, 2017.

Seizure-induced cardiac arrhythmias manifest in a wide range of clinical phenotypes, including tachycardia, bradycardia, atrioventricular-conduction block, atrial flutter and ventricular fibrillation [4–6]. Among these, seizure-induced sinus arrhythmias, e.g., sinus tachycardia, bradycardia, or asystole during ictal and postictal phases [6,10–12], are frequently observed and may act as potential triggers for SUDEP or occur around the same time as SUDEP events [13–15]. While ictal tachycardia can occur in up to 80% of seizures, ictal bradycardia or asystole are rarer events and may cause syncope and subsequent falls [6]. Intriguingly, previous clinical studies have documented that ictal tachycardia following the onset of seizures may progress into bradycardia and even prolonged asystole (Fig 1A) [16–18]. This puzzling transition from ictal tachycardia to bradycardia and asystole has been attributed to a neurogenic mechanism involving an initial surge in sympathetic nervous activity (SNA), followed by a transient increase in parasympathetic nervous activity (PNA) and a dramatic decrease in sympathetic tone originating from the cardiovascular center in the medulla due to prolonged seizures [16,17,19–21]. In addition to this ictal pattern, Al-Aweel *et al* [22] have identified another piece of this puzzle during the postictal phase. They observed transient but prominent postictal low-frequency heart rate oscillations (0.01 to 0.1 Hz) in patients with partial epilepsy (Fig 1B), suggesting that this postictal phenomenon could indicate neuroautonomic instability [22]. However, it remains unclear whether these seizure-induced ictal and postictal patterns depend solely on neurogenic mechanisms or if other contributing factors can be involved [23,24]. Besides the autonomic nervous system (ANS), cardiac pacemaking activities can be influenced by the intrinsic properties of the sinoatrial nodal cells (SANCs). For example, non-linear interactions between membrane and intracellular Ca oscillations of SANCs may drive changes in heart rates under pathological conditions [25,26]. Moreover, the interplay between the slow and fast dynamics of SANCs can result in abnormal cardiac pacemaking patterns [27].

Recent studies have begun to identify not only seizure-related cardiac alterations but also the correlation of circadian rhythm and sleep-wake patterns with seizures, which may impact SUDEP prevalence [29–32]. Previous studies suggest that day-night differences in seizure severity and susceptibility to SUDEP may be attributed to central circadian rhythms influencing neuronal excitability and regulating cardiac function through the ANS, thereby predisposing both the brain and heart to epileptic seizures and SUDEP [33–36]. However, recent evidence indicates that local circadian rhythmicity (LCR) in the heart may also contribute to these differences [37,38]. Day-night variations in hyperpolarization-activated cyclic nucleotide-gated (HCN) channel expression in SANCs are critical for cardiac pacemaking function and could be associated with cardiac arrhythmias under pathological conditions [39]. This link is further supported by experimental studies suggesting that cardiac HCN channelopathy can develop in rat epilepsy models [37]. Interestingly, experimental studies report a higher incidence of seizure-induced death in mice during sleep, regardless of circadian phases, suggesting that sleep-wake patterns might be a stronger indicator of seizure-induced death than circadian patterns (Fig 1C) [35]. Despite these findings, the mechanisms responsible for the disparity between circadian and sleep-wake patterns of seizure-induced deaths remain elusive.

**Fig 2:**
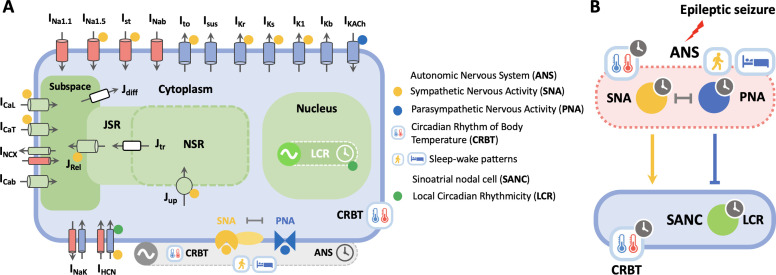
Schematic of a mouse SANC model incorporating circadian and vigilance state variations. **(A)** Each heartbeat is initiated by an action potential (AP) generated in a SANC. The firing patterns of SANC APs are shaped over a 24-hour cycle by circadian variations in the ANS, body temperature (BT), and LCR (green dot). The ANS, regulated by the master circadian clock located in the suprachiasmatic nucleus, finely adjusts the balance between SNA (yellow dot) and PNA (blue dot). This autonomic balance modulates SANC firing rates (FR) via SNA (e.g., I_HCN_, L-type (I_CaL_) and T-type Ca currents (I_CaT_)) and PNA-dependent (e.g., muscarinic K current (I_KACh_)) regulatory targets, adapting to diverse physiological demands throughout the day. The circadian rhythm of body temperature (CRBT) further regulates this balance, impacting the kinetics and/or conductance of ion channels, exchangers, and pumps in a SANC. Moreover, vigilance state (sleep/wake) changes, e.g., high PNA during sleep and low PNA during wakefulness, also modulate the autonomic balance in the mouse SANC. **(B)** During an epileptic seizure event, the well-maintained autonomic balance can be disrupted, potentially leading to distinct ictal and postictal outcomes, influenced by both circadian and vigilance state variations. The CRBT icon is derived from https://openclipart.org/detail/231080/thermometer, while the circadian clock icon is modified based on https://openclipart.org/detail/198766/mono-tool-timer.

To unravel these questions, we dissect the role of neurogenic and cardiogenic mechanisms underlying seizure-induced sinus arrhythmias using a SANC model (Fig 2) [26]. Our findings reveal that varying autonomic levels between ictal and postictal phases, while keeping within-phase autonomic activity constant, can trigger diverse seizure-induced SANC firing patterns, such as low-frequency oscillations in SANC firing rates (FR) and transitions from ictal tachycardia to bradycardia and asystole. Our results suggest that neurogenic mechanisms primarily drive across-phase SANC firing patterns, while cardiogenic mechanisms are responsible for within-phase firing patterns. This challenges the conventional view that attributes these outcomes solely to within-phase changes in autonomic balance. Our neurocardiogenic framework successfully captures distinct circadian patterns of seizure-induced sinus arrhythmia in mice. Daytime sleep predisposes SANC to postictal sinus arrhythmia, whereas nighttime wakefulness tends to promote ictal sinus arrhythmia. However, these circadian patterns can be disrupted when sleep-wake cycles are decoupled from circadian rhythms, supporting the hypothesis that sleep-wake patterns appear to be a more critical determinant of seizure-induced sinus arrhythmias. Our model simulations provide novel insights that could inform the development of chronobiological management and prevention strategies for seizure-induced deaths or SUDEP.

## Results

### Transient autonomic changes are not required to trigger ictal and postictal sinus arrhythmias

**Fig 3:**
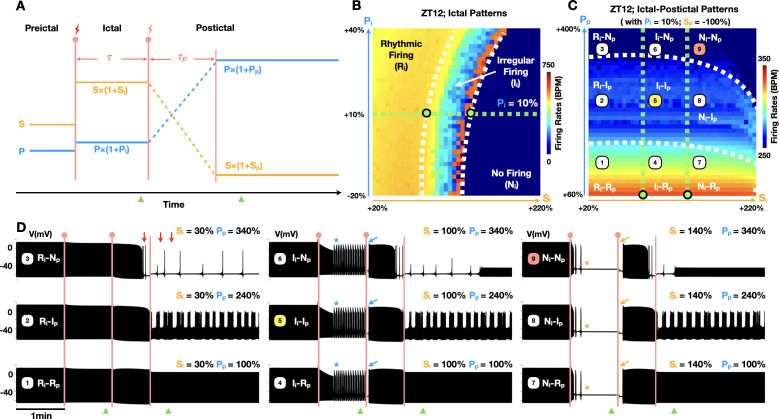
Transient autonomic changes are not required to trigger ictal and postictal sinus arrhythmias. **(A)** Illustration of the autonomic clamping protocol used to induce epileptic seizures in the SANC model without within-phase autonomic changes. A seizure event with the duration of τ is simulated by a dominant level of ictal SNA (S×(1 + S_i_)) over ictal PNA (P×(1 + P_i_)) (S_i_ > P_i_). After such an event, postictal levels of SNA and PNA are linearly ramped towards S×(1 + S_p_) and P×(1 + P_p_), respectively, with a ramping duration of τ_p_. Green triangles indicate the locations where ictal and postictal SANC FR are sampled. **(B-C)** Resulting ictal **(B)** and ictal-postictal parameter space maps **(C)** at the sleep-wake transition phase (light off at zeitgeber time (ZT) 12 in a 12h:12h lighting regime [41]). **(B)** When S_i_ and P_i_ are varied, the borders between rhythmic (R_i_), irregular (I_i_), and no-firing (N_i_) regions of ictal SANC firing patterns are indicated by white dashed lines. **(C)** After setting P_i_ to 10% (B; green dashed line and dots) and S_p_ to -100%, S_i_ and P_p_ are varied to obtain the postictal parameter space map. Green and white dashed lines delineate the borders between rhythmic (R), irregular (I), and no-firing (N) regions of ictal (i) and postictal (p) SANC firing patterns, respectively. This results in a total of nine distinct regions in the parameter space map (labeled 1 to 9). **(D)** Simulation traces of SANC membrane potentials (black traces) sampled from the nine parameter regions, demonstrating a wide range of seizure-induced SANC excitation patterns. These include ictal sinus rhythm [1–3], mixed ictal tachycardia and bradycardia [4–6], ictal asystole [7–9], postictal sinus rhythm [1,4,7], postictal low-frequency oscillations [2,5,8] and postictal asystole [3,6,9]. Simulation traces labeled 5 (yellow; I_i_-I_p_) and 9 (red; N_i_-N_p_) depict oscillatory and asystolic seizure events, respectively.

To dissect neurocardiogenic mechanisms underlying seizure-induced sinus arrhythmias, we implemented an autonomic clamping protocol — a computational method that specifies the levels of sympathetic and parasympathetic inputs over prescribed time windows (Fig 3A) — in our recently developed mouse SANC model (Fig 2A) [26]. By eliminating autonomic fluctuations during and after seizure events, this approach isolates intrinsic cardiogenic factors contributing to arrhythmias. Specifically, for a typical seizure event lasting a duration of τ (Fig 3A), preictal (before seizure) SNA (S) and PNA (P) are adjusted by their ictal scaling factors, S_i_ and P_i_, respectively, to simulate a seizure-induced shift in autonomic balance as seen in [40]. During the seizure, SNA and PNA are promptly clamped to their ictal values, S×(1 + S_i_) and P×(1 + P_i_), respectively, for the duration of τ (Fig 3A). Considering that ictal tachycardia may occur in up to 80% of seizures [6], we assume an ictal sympathetic dominance over the parasympathetic system, so that S_i_ > P_i_. Following the seizure, SNA and PNA linearly ramp towards their postictal values, S×(1 + S_p_) and P×(1 + P_p_), respectively, over a ramping duration of τ_p_ (Fig 3A). Here, we assume a postictal parasympathetic dominance (P_p_ > P_i_) with a minimal SNA (S_p_ = -100%), potentially allowing the recovery of sinus rhythms from ictal sinus arrhythmias. It is important to note that the epileptic parameters, namely, S_i_, P_i_, S_p_, P_p_, τ, τ_p,_ remain actively operative within the model under autonomic clamped conditions, allowing state variables to freely evolve and manifest seizure-induced SANC dysfunction. Thus, while autonomic clamping stabilizes external inputs, the epileptic parameters and their impact on model state variables remain necessary to disrupt SANC function.

Using this autonomic clamping protocol, we first simulated the ictal S_i_-P_i_ parameter space map (Fig 3B) —a heatmap-based approach that examines how different combinations of two model parameters influence SANC activity patterns. To this end, we varied S_i_ from 20% to 220% and P_i_ from -20% to 40% and measured the ictal SANC FR in beats per minute (BPM) at the end of the seizure window (Fig 3A; left green triangle). As S_i_ increases, the ictal SANC activity pattern can degenerate from rhythmic firing (R_i_) to irregular firing (I_i_), and finally to no firing (N_i_) (Fig 3B). On the other hand, increasing P_i_ expands the region of rhythmic firing (R_i_), reducing the extent of the no firing region (N_i_) (Fig 3B). Interestingly, the transition from rhythmic firing to irregular firing and ultimately to no firing proceeds along the horizontal axis (from left to right), highlighting the dominant influence of Sᵢ in shaping ictal firing patterns of SANC.

Next, we simulated the ictal-postictal S_i_-P_p_ parameter space map (Fig 3C) to characterize SANC activity patterns both during and after seizures. For this step, we varied S_i_ from 20% to 220% and P_p_ from 60% to 400%, while fixing P_i_ at 10% (Fig 3B; green dashed lines and dots) and S_p_ at -100%. Then, we sampled the SANC FR both before and after the postictal ramp (Fig 3A; left and right green triangles). These simulations yielded a S_i_-P_p_ parameter space map segmented into nine distinct regions, delineated by green dashed lines (ictal patterns) and white dashed lines (postictal patterns) in Fig 3C. These regions correspond to rhythmic (R), irregular (I), or no firing (N) patterns of ictal (i) and postictal (p) SANC activity. In contrast to the horizontal transition observed in Fig 3B, a mostly vertical transition (from bottom to top; Fig 3C) across these firing regions is evident, underscoring the critical role of P_p_ in shaping postictal dynamics.

From each of these nine regions (labeled 1–9 in Fig 3C), we sampled simulation traces of SANC membrane potentials (Fig 3D) to demonstrate the diverse and dynamic changes of SANC activity both during and after the seizure event. Specifically, when S_i_ is low (traces 1–3; Fig 3D), ictal SANC excitation remains rhythmic, while postictal SANC activity can degenerate from a sinus rhythm to low-frequency FR oscillations and asystole with increasing P_p_. Notably, postictal asystole can be observed both during and after the postictal ramp (red arrows, trace 3; Fig 3D) when P_p_ is high. With moderately increased S_i_ (traces 4–6; Fig 3D), postictal SANC excitation patterns remain mostly unchanged compared to low S_i_ (traces 1–3; Fig 3D); however, ictal low-frequency oscillations between tachycardic and bradycardic states can be observed in the SANC FR (blue stars, traces 4–6; Fig 3D). Interestingly, transient events of postictal sinus arrest can be observed immediately after seizure events (blue arrows, traces 4–6; Fig 3D). With a high S_i_ (traces 7–9; Fig 3D), mixed ictal tachycardia and bradycardia can further degenerate into ictal asystole after the onset of seizure events (yellow stars, traces 7–9; Fig 3D), accompanied by prolonged events of postictal sinus arrest (yellow arrows, traces 7–9; Fig 3D).

Interestingly, our simulation results (traces 7–9; Fig 3D) suggest that even when ictal sympathetic and parasympathetic tones were clamped with no dynamic changes during the seizure (Fig 3A), a neurocardiogenic mechanism can drive transitions from ictal tachycardia to bradycardia, and ultimately asystole (Fig 1A). This contrasts with the neurogenic mechanism [17], which attributes ictal bradycardia and asystole to a transient increase in vagal tone during the seizure. Furthermore, we also found that both ictal (traces 4–6; Fig 3D) and postictal (traces 2, 5, 8; Fig 3D) low-frequency oscillations in SANC FR (Fig 1B) can emerge with clamped autonomic activity, suggesting a neurocardiogenic explanation that does not rely on autonomic instability [22].

### Neurocardiogenic mechanisms underlying seizure-induced sinus arrhythmias

**Fig 4:**
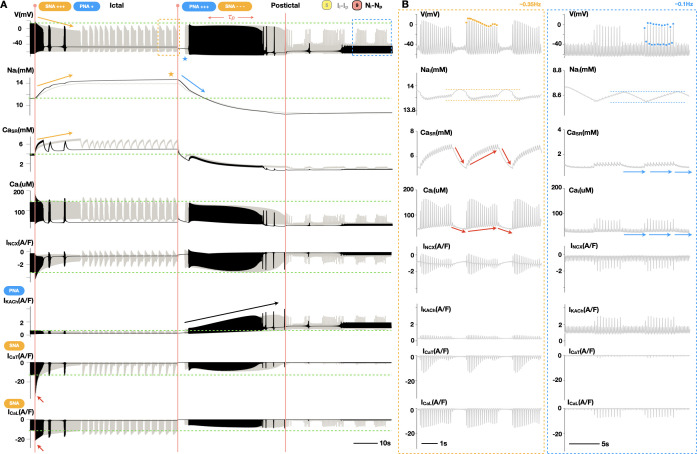
Neurocardiogenic mechanisms underlying seizure-induced arrhythmias in SANC excitation patterns. **(A)** Detailed electrophysiologic and ionic dynamics behind representative seizure events featuring ictal and postictal low-frequency oscillations (trace 5 from Fig 3C and 3D; grey traces) and asystole (trace 9 from Fig 3C and 3D; black traces). **(B)** A close-up comparison between ictal (A; yellow dashed box) and postictal (A; blue dashed box) clusters of bursting APs with underlying intracellular Na, Ca dynamics, and key ionic currents in trace 5 (A; grey).

To investigate the neurocardiogenic mechanisms underlying distinct patterns of seizure-induced SANC activity, we compared the electrophysiological and ionic details behind representative oscillatory (trace 5; Fig 3D) and asystolic (trace 9; Fig 3D) seizure events (grey (trace 5) and black (trace 9) traces; Fig 4A). Specifically, during the seizure, SANC FR promptly increases due to the acute effects of SNA dominance over PNA, i.e., enhanced I_CaT_ and I_CaL_ (red arrows; Fig 4A). As the seizure progresses, this elevated FR allows more Na to enter the cell, leading to intracellular Na accumulation, Ca overload, and a subsequent reduction in SANC excitability (yellow arrows; Fig 4A), attributed to enhanced Ca dependent inactivation of I_CaL_. In the steady-state phase, a balance is reached between the opposing effects of heightened SNA and intracellular Na accumulation, ultimately determining the ictal SANC firing patterns, i.e., ictal low-frequency oscillations (grey; Fig 4A) or, when these effects are even stronger, asystole (black; Fig 4A). After the seizure, PNA dominates over SNA, resulting in intracellular Na depletion from its ictal levels (blue arrow; Fig 4A). Compared to trace 5 (grey; Fig 4A), a prolonged postictal sinus arrest (blue star; Fig 4A) can be observed in trace 9 (black; Fig 4A) due to higher Na loading during the seizure (yellow star; Fig 4A). Further reduction in Na content can restore sinus rhythm. However, such restoration can be transient (Fig 4A) due to increasing postictal PNA, which reduces SANC excitability by enhancing I_KACh_ (black arrow; Fig 4A). After the postictal ramp (τ_p_), SANC excitability is co-modulated by steady-state SNA and PNA levels and is mechanistically determined by intracellular Na content and I_KACh_. This results in postictal SANC firing patterns such as low-frequency oscillations (grey; Fig 4A) or asystole (black; Fig 4A).

However, it is evident that compared to their ictal counterparts (yellow dashed box; Fig 4A), postictal low-frequency oscillations in SANC excitation (blue dashed box; Fig 4A) are characterized by their distinct AP morphologies, frequencies, and intracellular ionic profiles. Thus, we further investigated electrophysiologic and ionic mechanisms underlying the differences between ictal and postictal bursts of APs (Fig 4B). Specifically, ictal bursts of APs (~0.35Hz) (yellow dots; Fig 4B) have higher amplitudes, while beat-to-beat AP alternans can be observed in postictal bursts of APs (~0.1Hz) (blue dots; Fig 4B). Both ictal and postictal bursts of APs involve cyclical Na fluctuations of similar amplitudes but they operate at high (~13.9mM) (yellow dashed lines; Fig 4B) and low (~8.6mM) (blue dashed lines, Fig 4B) Na levels, respectively. Postictal Na fluctuations are mostly linear (blue dashed lines, Fig 4B), with Na accumulating during bursts of APs and declining between bursts, while ictal Na fluctuations are more non-linear (yellow dashed lines; Fig 4B), with a rise in sodium that continues after the offset of each burst. These ictal sodium effects relate to the pronounced Ca contributions to ictal oscillations (red arrows; Fig 4B). Indeed, while postictal oscillations in intracellular Ca are minimal and evoke only weak I_CaL_ and negligible I_CaT_ (blue arrows; Fig 4B), the distinct levels of ictal autonomic signals result in large contributions of both I_CaT_ and I_CaL_ to ictal bursts. The resulting elevation of intracellular Ca boosts activity of sodium-calcium exchanger current_,_ which pumps calcium out of the SANC in between bursts of APs while helping to prolong the elevation of the intracellular Na concentration during those periods.

### Circadian and vigilance state regulation of seizure-induced sinus arrhythmias

**Fig 5:**
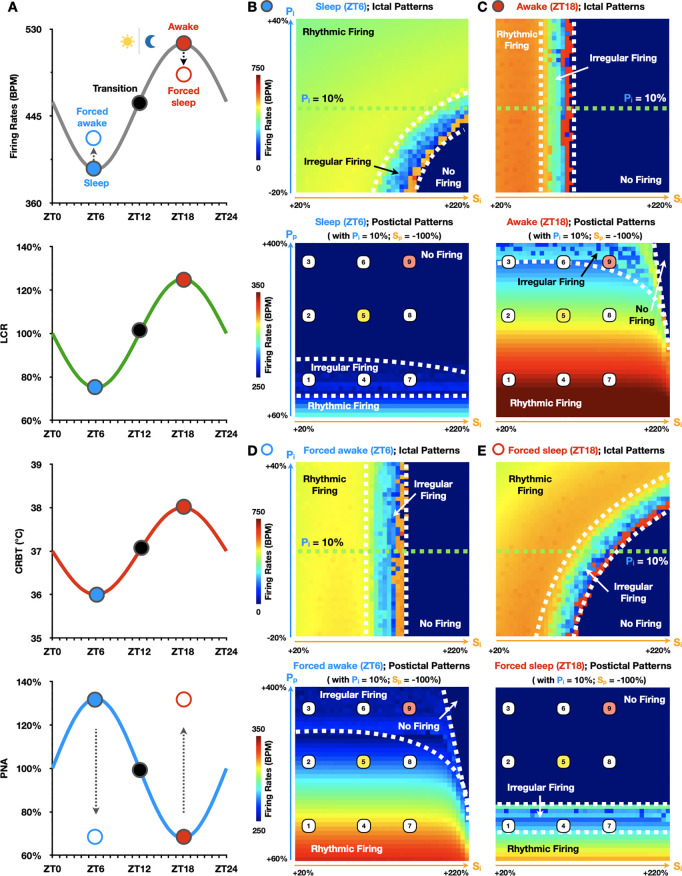
Regulation of seizure-induced SANC firing patterns by circadian rhythms and vigilance states. (A) Simulated circadian rhythms of SANC FR (grey curve), LCR (green curve), CRBT (red curve), and PNA (blue curve) over a 24-hour cycle under a 12h:12h lighting regime. SANC FR, LCR, and CRBT peak at ZT18 during nighttime wakefulness (awake state; solid red dot), and hit bottom at ZT6 during daytime sleep (sleep state; solid blue dot). In contrast, PNA peaks at ZT6 and has a minimum at ZT18. The solid black dot represents the transition state (at ZT12). Red and blue empty dots indicate the forced sleep (at ZT18) and forced awake (at ZT6) states with off-phase PNA, respectively. (B-C) Differences in ictal (top) and postictal (bottom) parameter space maps between the sleep (B) and awake (C) states. (D-E) Ictal (top) and postictal (bottom) parameter space maps when circadian rhythms are decoupled from sleep-wake patterns via forced awake (D) and forced sleep (E) protocols.

These neurocardiogenic insights (Fig 4) add a critical dimension to our understanding of the brain-heart interactions underlying seizure-induced sinus arrhythmias. However, they were investigated only at the transition state (ZT12) between sleep and wake (black dots; Fig 5A), assuming no variations in circadian and vigilance state conditions, i.e., no time-of-day differences in autonomic balance, BT, LCR and sleep/awake patterns (Fig 2). To quantitatively study circadian and vigilance state regulation of seizure-induced sinus arrhythmias, we introduced four additional states into our model (blue and red dots; Fig 5A). Specifically, concurrently with naturally waking up during the night (awake state at ZT18; red dots, Fig 5A), SANC FR (grey curve; Fig 5A) peaks due to high expression levels of I_HCN_ driven by LCR (green curve; Fig 5A), high BT (red curve; Fig 5A), and low PNA (blue curve; Fig 5A). Conversely, during daytime sleep (sleep state at ZT6; blue dots, Fig 5A), SANC FR bottoms out due to low expression levels of I_HCN_, low BT, and high PNA. Here, we assumed minimal circadian variations in SNA, which may be attributed to a high baseline sympathetic tone in mice to maintain a normal core temperature (37°C) under standard laboratory conditions (20°C), potentially limiting additional time-of-day fluctuations as previously reported [26]. In addition to these circadian states (solid dots; Fig 5A), vigilance states, i.e., forced sleep and awake states (red and blue empty dots; Fig 5A), were introduced to decouple circadian rhythms from sleep-wake patterns. Specifically, these vigilance states were phenomenologically modeled by assuming unchanged LCR and BT with off-phase PNA at ZT18 and ZT6, respectively.

Next, we generated parameter space maps with these circadian and vigilance state variations (blue and red dots; Fig 5A), using the same simulation protocols described earlier (Fig 3B and 3C). Compared to the transition state at ZT12 (Fig 3B and 3C), during daytime sleep (Fig 5B), the no-firing region shrinks in the ictal parameter space map but expands in the postictal parameter space map. In addition, the ictal transition from rhythmic firing to irregular firing to no-firing regions becomes diagonal (from top left to bottom right) in the parameter space map (Fig 5B; upper panel), in contrast to the mostly horizontal transition observed in Fig 3B. This pattern suggests a more balanced contribution from S_i_ and P_i_ in driving ictal SANC excitations during daytime sleep. Conversely, after waking up at ZT18 (Fig 5C), the no-firing region expands in the ictal parameter space map yet shrinks in the postictal parameter space map, relative to ZT12 (Fig 3B and 3C). Notably, the ictal transition across firing regions becomes almost perfectly horizontal, indicating a minimal contribution of P_i_ in determining ictal SANC firing patterns during wakefulness.

Interestingly, when circadian rhythms are decoupled from sleep-wake patterns (Fig 5D and 5E), forced wakefulness at ZT6 (Fig 5D) results in an expansion of the no-firing region in the ictal map, and a reduction in the postictal map compared to natural sleep at ZT6 (Fig 5B). In this condition, the ictal transition from rhythmic firing, irregular firing to no firing regions becomes fully horizontal in the parameter space map (Fig 5D; upper panel), confirming that P_i_ has little influence in driving ictal excitation patterns during forced wakefulness. Conversely, forced sleep at ZT18 (Fig 5E) results in a reduced no-firing region in the ictal map and an expansion in the postictal map compared to natural wakefulness at ZT18 (Fig 5C). Under forced sleep, the ictal transition across firing regions shifts from horizontal to diagonal in the parameter space map, suggesting a balanced contribution of P_i_ and S_i_ in shaping ictal SANC firing patterns.

**Fig 6:**
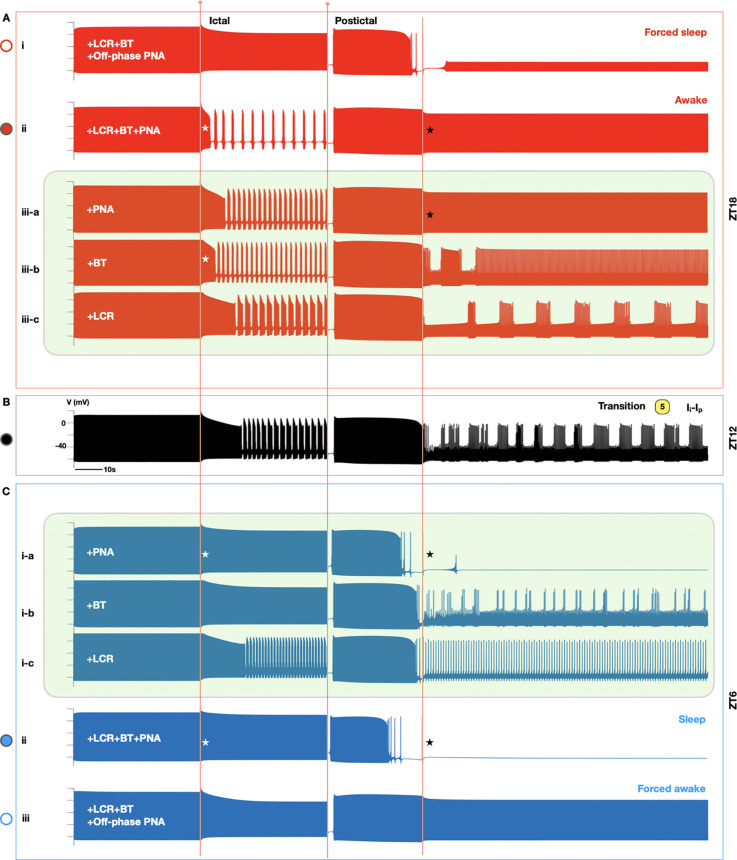
Dissecting key mechanisms governing circadian and vigilance state regulation of seizure-induced SANC firing patterns. Representative oscillatory (labeled as 5 in Fig 5B–E) seizure events under different circadian (at ZT18 (A), ZT12 (B), and ZT6 (C)) and vigilance state (forced sleep at ZT18 (A-i) and forced awake at ZT6 (C-iii)) conditions. Time-of-day changes in PNA (A-iii-a; ZT18) (C-i-a; ZT6), CRBT (A-iii-b; ZT18) (C-i-b; ZT6), and LCR (A-iii-c; ZT18) (C-i-c; ZT6) were applied individually to dissect the relative contributions of each circadian factor in shaping distinct SANC firing patterns at ZT18 (upper green box) and ZT6 (lower green box), respectively.

To dissect and identify key mechanisms responsible for these changes in parameter space maps (Fig 5B–E), we compared simulated traces of seizure-induced membrane excitations during an oscillatory seizure event (labeled as trace 5 in Figs 3C and 5B–E) under various circadian and vigilance state conditions (Fig 6). Specifically, ictal low-frequency oscillations in SANC firing patterns at ZT12 (Fig 6B) may escalate at ZT18 (ii; Fig 6A), but are suppressed at ZT6 (ii; Fig 6C). Conversely, postictal low-frequency oscillations at ZT12 (Fig 6B) can normalize to sinus rhythm at ZT18 (ii; Fi 6A) or degenerate to asystole at ZT6 (ii; Fig 6C). To identify key mechanisms underlying these circadian patterns in ictal and postictal SANC activity, each circadian factor was applied individually to determine its relative contribution in shaping seizure-induced SANC patterns (green boxes; Fig 6). Notably, circadian changes in PNA (i-c; Fig 6C) dominate over other circadian factors in determining both ictal (white stars; Fig 6C) and postictal (black stars; Fig 6C) SANC firing patterns at ZT6 (ii; Fig 6C). However, at ZT18, circadian changes in BT (iii-b; Fig 6A) are most important in promoting ictal low-frequency oscillations (white stars; Fig 6A), while circadian changes in PNA remain the dominating factor in restoring postictal sinus rhythm (black stars; Fig 6A). Interestingly, when circadian rhythms are decoupled from sleep-wake patterns, our simulations suggest that postictal asystole during sleep can occur regardless of the time of day (i; Fig 6A versus ii; Fig 6C). This finding is in agreement with experimental findings (Fig 1C) [6,28] and suggests that sleep-wake patterns may be a stronger indicator of seizure-induced deaths or SUDEP compared to circadian variations.

## Discussion

In this study, we examined the critical yet largely overlooked neurocardiogenic mechanisms that contribute to seizure-induced sinus arrhythmias using a mathematical model of mouse SANC [26] (Fig 1A–B). Epileptic seizure events were simulated by varying autonomic levels between the ictal and postictal phases. To eliminate the impact of transient autonomic changes during these phases, we implemented an autonomic clamping protocol with SNA and PNA clamped at fixed levels. Our results reveal that intrinsic cardiogenic mechanisms alone can suffice to drive ictal and postictal low-frequency oscillations as well as the transition from ictal tachycardia to bradycardia and asystole (Figs 3 and 4). These findings suggest distinct roles for neurogenic and cardiogenic mechanisms in shaping across-phase and within-phase SANC firing patterns, respectively. Our results underscore the role of cardiogenic factors in seizure-induced sinus arrhythmias, contrasting with the neurogenic mechanism that attributes these arrhythmias to temporally varying autonomic signals during or after seizures [17,22].

Additionally, we investigated the impact of circadian rhythms and vigilance states on seizure-induced sinus arrhythmias. Both clinical and experimental recordings have reported circadian patterns in seizure-induced deaths and SUDEP; however, a high fatality rate is observed for maximal electroshock-induced seizures occurring during sleep, regardless of the circadian phase (Fig 1C) [28]. Our simulation results highlighted the role of PNA in shaping both circadian and vigilance state patterns of postictal sinus arrhythmias, frequently linked to SUDEP or near SUDEP events (Fig 6). Specifically, we found that postictal asystole can occur during sleep regardless of the time of day (Fig 6), suggesting that sleep-wake patterns can be a stronger indicator of seizure-induced deaths or SUDEP than circadian variations [29]. This finding is in agreement with both experimental data (Fig 1C) and clinical evidence [42]. For example, clinical studies indicate that sleep-wake patterns are stronger predictors of pediatric seizures compared to circadian rhythm [43,44]. Seizure occurrences in frontal lobe epilepsy are more closely aligned with transitions between sleep and wakefulness, both during the day and night [45]. In addition, our simulations indicate that being vigilant during the postictal phase may help prevent SUDEP (Fig 5D). A case-control study supports this finding that having someone present at night reduces the likelihood of SUDEP (odds ratio 0.4) [46]. This intervention involves family members observing seizures that occur at bedtime and then waking the individual during the postictal phase [47]. Moreover, our simulation results suggest that circadian changes in BT play a significant role in promoting ictal sinus arrhythmias (Fig 6A). Intriguingly, patients with Dravet syndrome exhibit a disproportionately high incidence of sudden death in bathtubs, often in the absence of drowning evidence [48,49]. Hot water immersion, which triggers sympathetic activation and raises BT, increases seizure risk and amplifies SUDEP susceptibility within this vulnerable population [49–51].

These neurocardiogenic and chronobiological insights could potentially inform the development of therapeutic strategies aimed at managing seizure-induced cardiac arrhythmias and mitigating risks associated with SUDEP. For example, antiepileptic drugs like lamotrigine may increase the risk of SUDEP [52]. Recently, the US Food and Drug Administration issued a safety warning on the cardiac effects of lamotrigine, highlighting its potential to slow ventricular conduction and promote sudden death [53]. Notably, elevated heart rates, such as those occurring during the ictal phase of a seizure event (Figs 3 and 4), could further exacerbate the risk of slowed ventricular conduction with lamotrigine. Future research may utilize the model and approaches developed in this study to identify the ionic mechanisms underlying the adverse effects of lamotrigine in promoting SUDEP and cardiac arrhythmias.

Furthermore, chronotherapy, i.e., the strategic timing of drug administration to optimize efficacy while minimizing side effects [54–58], has been applied in treating cancer and cardiovascular diseases [59,60]. Currently, drug-resistant epilepsy affects one-third of patients and is associated with an increased risk of SUDEP [61], and chronotherapy could offer an opportunity to reduce the likelihood of drug-resistant epilepsy [62]. For example, clinical studies have suggested that epileptic patients who took the majority of their antiepileptic drugs at 8pm experience better seizure control and drug tolerance compared to those on a twice-daily dosing regimen [62]. Interestingly, our simulation results (Fig 6) indicate that sleep-wake patterns may be more predictive of seizure-induced deaths or SUDEP than circadian variations. The efficacy of chronotherapeutic interventions may therefore be influenced by an individual’s sleep-wake cycles, possibly due to elevated PNA during sleep. Two complications in the design of such treatments are that up to two thirds of epileptic patients experience sleep disturbances [63] and that antiepileptic drugs can disrupt sleep architecture, leading to side effects such as insomnia or fragmented sleep [64]. These disruptions may further exacerbate seizure activity by altering brain excitability and reducing drug efficacy, creating a vicious cycle: antiepileptic drug-induced sleep disturbances impair seizure control, potentially necessitating higher or additional dosing, which in turn further compromises sleep [65]. In light of these complexities, the model developed in our study can serve as a valuable tool for the identification of optimal drug targets and chronotherapeutic dosing strategies, offering a theoretical basis for personalized interventions tailored to mitigate SUDEP risk in patients with drug-resistant epilepsy.

Moreover, it should be noted that, in addition to neurogenic and cardiogenic factors, respiratory mechanisms may also contribute to the development of SUDEP [13], complicating the design of effective treatments for patients with epilepsy. For example, clinical observations in patients with epilepsy reveal a surprising distinction: while ictal asystole events in these patients are often self-limiting [17,18], postictal asystole events are frequently linked to SUDEP or near SUDEP events [6]. This pattern may derive from the complex neuro-cardio-respiratory interactions during the ictal and postictal phases [13]. For instance, our simulation results demonstrated that both ictal and postictal asystole can occur, yet they are characterized by unique electrophysiological profiles and intracellular Na concentrations in SANCs (Fig 4). Here, we speculate that during the ictal phase, the negative respiratory feedback could terminate the seizure event, and release SANC from its ictal condition of excessive Na content, potentially allowing the recovery of sinus rhythm. However, during the postictal phase with a low Na content in SANC, the respiratory feedback can only further impair SANC excitability, promoting the development of SUDEP. Thus, the development of a more integrated systems model that quantitatively describes the delicate interactions among neural, cardiac, and respiratory systems holds great potential for the identification of novel drug targets and chronotherapeutic strategies to improve seizure management and reduce SUDEP risk.

In addition to the complex neuro-cardio-respiratory interactions during the ictal and postictal phases, which typically last minutes, the severity of a seizure event can be influenced by chronic remodeling in epilepsy that develops over a much longer period, e.g., months or years [66,67]. For instance, the risk of SUDEP is 20–40 times higher in patients with chronic epilepsy, compared to the general population. Chronic epilepsy is associated with dysfunction in the ANS, disruption of the respiratory system, and permanent alterations in cardiac excitation patterns, e.g., heightened heart rates and prolonged QT intervals. Specifically, as a chronic consequence of epileptic seizures, the emergence of cardiac HCN channelopathies [37] may predispose the heart to the development of cardiac arrhythmias, potentially due to persistent aberrant autonomic inputs to the heart [66]. Within this context, a minor seizure event (trace 1; Fig 3D) may potentially escalate to more severe event lethal outcomes (trace 5 or 9; Fig 3D), depending on the progression of chronic remodeling in epilepsy. Thus, chronic remodeling in neuro-cardio-respiratory systems can be a crucial dimension for establishing an integrative pathophysiological understanding of SUDEP.

Our study has several limitations that may impact the interpretation of our model simulations. Our model is developed to be mouse-specific, which necessitates careful considerations when extrapolating our results to humans due to cross-species differences [68]. For example, humans are diurnal, with a resting heart rate of approximately 60–100 BPM, whereas mice are nocturnal and exhibit a much higher basal heart rate of around 500–600 BPM [69,70]. Moreover, human sleep architecture is typically monophasic or biphasic, characterized by one or two primary sleep episodes at night, while mice display polyphasic sleep, consisting of multiple short bouts distributed across the 24-hour cycle [71]. These species-specific differences are further amplified by variations in intrinsic autonomic tone and responsiveness between mice and humans [41]. Despite these distinctions, our mouse model of SANC successfully reproduced a wide spectrum of seizure-induced arrhythmic patterns observed in humans [6], including sinus tachycardia, bradycardia, and asystole (Fig 3D). Importantly, without introducing within-phase autonomic fluctuations, our simulations recapitulated the dynamic progression from ictal tachycardia to bradycardia and eventually to asystole, along with the emergence of postictal low-frequency oscillations, as reported in epileptic patients (Fig 1A and 1B) [16,22]. In addition, our simulation results closely align with experimental findings from mouse models of epileptic encephalopathy and SUDEP [72,73]. For example, both cardiac and parasympathetic hyperactivity have been implicated in contributing to SUDEP in *Scn8a*^*N1768D/+*^ mice [72], consistent with our findings on the critical role of PNA in shaping postictal SANC activity patterns (Fig 3C and 3D). Furthermore, *Kv1.1* potassium channel null mice exhibit spontaneous seizure-induced sinus arrhythmias and SUDEP events exclusively following seizures [73], supporting our simulation results that postictal sinus bradycardia may facilitate the development of SUDEP, potentially attributed to low intracellular Na content in SANCs (Fig 4).

We introduced forced sleep and awake states with off-phase PNAs, which may oversimplify the complex physiological and metabolic aspects associated with sleep [74]. We did not separately study the effects of rapid eye movement sleep and non-rapid eye movement sleep, due to limited data availability to characterize these two sleep patterns for our study [28]. Additionally, while the autonomic clamping protocol effectively identified neurocardiogenic mechanisms in seizure-induced sinus arrhythmias, real-world scenarios are far more complex, driven by dynamic neurocardiac excitation patterns and their bidirectional coupling during both ictal and postictal phases [75]. For example, seizures can disrupt brain regions that regulate autonomic output, leading to rapid shifts that may accelerate the development of cardiac arrhythmias. Conversely, cardiac disturbances can reciprocally influence brain function by impairing cerebral perfusion, disrupting autonomic feedback loops, and causing hypoxia [76]. This bidirectional interaction can create either a protective or pathological cycle, in which neurological instability alters cardiac function, and cardiac dysfunction either mitigates or exacerbates neurological impairment. The integration of dynamic neurocardiac interactions into our model may potentially delineate the relative contributions of neurogenic versus cardiogenic mechanisms in driving seizure-induced sinus arrhythmias, e.g., by using a state-variable clamp protocol [77].

In our model, autonomic activity levels, i.e., SNA and PNA, are represented as a set of model parameters that map to changes in ion channel properties, such as channel conductances, under varying levels of sympathetic and parasympathetic tone. Similar model behaviors to those observed in Fig 3D may be reproduced with a different or reduced set of model components, such as the interplay between I_CaL_ (a major SNA target) and I_KACh_ (the primary PNA target), raising the possibility that investigation of reduced models could yield specific predictions about the ionic mechanisms underlying seizure-induced sinus arrhythmias [78,79]. In this vein, from an analytical perspective, an interesting future direction would be to study the bifurcations in model dynamics induced by variation of parameters associated with model ion currents, perhaps aided by a timescale decomposition of model variables [80]. The relatively abrupt changes in dynamics seen upon conclusion of the autonomic ramp in some parameter regimes (Fig 3D) suggest the involvement of certain types of bifurcations, but careful analysis will be required to determine the details, which may involve a combination of delayed bifurcation [81] and slow dynamics effects. In future work, formal bifurcation analysis can be conducted on simplified versions of the model to identify specific transitions—such as Hopf, saddle-node on an invariant circle, or Neimark–Sacker bifurcations—and to establish links between these mathematically defined behaviors and their physiological correlates [82–84].

In addition, our study focused on investigating the cardiogenic mechanisms underlying seizure-induced sinus arrhythmias, specifically those originating from abnormal SANC firing patterns at the cellular level. However, the pacemaking activity of the sinoatrial node—a highly complex and heterogeneous three-dimensional structure [85]—is driven by the emergent behaviors of electrically coupled SANCs and their interactions with surrounding atrial tissue. These complexities at the tissue level, along with their modulation by circadian rhythms and sleep-wake cycles, may also contribute to cardiogenic mechanisms that were beyond the scope of this study. Future model development, including the systematic integration of neural excitation dynamics [75], respiratory feedback [86], chronic remodeling in epilepsy [66], and tissue-level complexities of sinoatrial nodal excitations [87–89] could pave the way for a deeper theoretical understanding of the complex brain-heart interactions in epilepsy, and provide a quantitative tool to accelerate the development of therapeutic strategies for the management and prevention of epilepsy and SUDEP.

## Materials and methods

Our recently developed mathematical model of the circadian regulation of mouse SANC pacemaking [26] was utilized for the computer simulations of SANC excitation presented in this study. All model definitions, equations, and parameter settings remain unchanged from those previously described [26]. In this model, SANC firing patterns are determined by the intricate interactions between a membrane oscillator, associated with membrane ionic currents, and a Ca oscillator driven by intracellular Ca cycling dynamics (Fig 2A), building upon the original work and model code implementations by Kharche *et al* [90] and Ding *et al* [91]. Importantly, both membrane and Ca oscillators are modulated by day-night rhythms in autonomic balance, BT, and LCR (Fig 2A), enabling the quantitative reconstruction of circadian patterns in SANC pacemaking function [26]. A 12h:12h light/dark lighting regime (Fig 5A) was implemented as in experimental studies [41].

All model simulations were performed using parallel computing on a ThinkStation P620 tower workstation with an AMD Threadripper processor. Model codes were implemented and solved in MATLAB (Version: 9.13.0 (R2022b)) using the ode15s solver. The model codes for computer simulations presented in this study are publicly available for download at https://github.com/Mathbiomed/SeizureSANC.

### Autonomic clamping

To induce epileptic seizure events in our SANC model, we implemented an autonomic clamping protocol without introducing transient changes in autonomic balance (Fig 3A). Specifically, during the preictal phase (60s), the model was simulated to achieve steady-state behaviors, using default initial conditions and parameter settings previously described [26]. At the onset of a seizure event, preictal SNA (S) and PNA (P) were promptly clamped to their ictal values, S×(1 + S_i_) and P×(1 + P_i_), respectively, for a duration of 60s (τ; Fig 3A). After the seizure event, ictal SNA (S×(1 + S_i_)) and PNA (P×(1 + P_i_)) linearly ramped towards their postictal values, S×(1 + S_p_) and P×(1 + P_p_), respectively, for a duration of 45s (τ_p_; Fig 3A). Following the ramp stage, the simulation continued for another 75s to achieve postictal steady-state behaviors. The epileptic parameters (i.e., S_i_, P_i_, S_p_, P_p_, τ, τ_p_) remain active under autonomic clamping, allowing seizure-induced SANC dysfunction to emerge from freely evolving state variables. The total time duration of a simulated epileptic seizure event was 4 minutes.

### Parameter space map

To identify potential seizure-induced SANC excitation patterns, we implemented a parameter space mapping protocol (Figs 3B, 3C and 5B–E). Specifically, the ictal parameter space map (Figs 3B and 5B–E, upper panels) was generated by varying S_i_ from 20% to 220% with a step size of 10%, and P_i_ from -20% to 40% with a step size of 1.5%, resulting in a total of 40 × 40 simulations per map, color-coded by ictal SANC FR. Here, the ictal SANC FR was sampled toward the end of the ictal phase (Fig 3A; left green triangle). The postictal parameter space map (Figs 3C and 5B–E, lower panels) was generated by varying S_i_ from 20% to 220% with a step size of 10%, and P_p_ from 60% to 400% with a step size of 8.5%, resulting in a total of 40 × 40 simulations per map, color-coded by postictal SANC FR. The postictal SANC FR was sampled after the postictal ramp (Fig 3A; right green triangle). For postictal maps (Figs 3C and 5B–E, lower panels), P_i_ and S_p_ were set to 10% and -100%, respectively.

### Circadian and vigilance state conditions

To investigate circadian and vigilance state regulation of seizure-induced sinus arrhythmias, we introduced 5 distinct model states to account for their variations. For circadian variations, the states included: the awake state at ZT18 (red dots; Figs 5 and 6), the transition state at ZT12 (black dots; Figs 5 and 6), and the sleep state at ZT6 (blue dots; Figs 5 and 6). For vigilance state variations, the states included: the forced sleep state at ZT18 (red empty dots; Figs 5–6) and the forced awake state at ZT6 (blue empty dots; Figs 5–6). Parameter settings for these circadian variations were kept the same as previously described [26]. For the forced sleep or awake states, we assumed off-phase circadian variations of PNA, with maximal PNA at ZT18 (instead of ZT6) and minimal PNA at ZT6 (instead of ZT18). This decouples the sleep-wake cycle from circadian rhythms while keeping other parameters unchanged [92].
